# Dietary Polyphenol Intake, but Not the Dietary Total Antioxidant Capacity, Is Inversely Related to Cardiovascular Disease in Postmenopausal Polish Women: Results of WOBASZ and WOBASZ II Studies

**DOI:** 10.1155/2017/5982809

**Published:** 2017-06-20

**Authors:** Anna M. Witkowska, Anna Waśkiewicz, Małgorzata E. Zujko, Danuta Szcześniewska, Andrzej Pająk, Urszula Stepaniak, Wojciech Drygas

**Affiliations:** ^1^Department of Food Biotechnology, Medical University of Bialystok, Bialystok, Poland; ^2^Department of Epidemiology, Cardiovascular Disease Prevention and Health Promotion, National Institute of Cardiology, Warsaw, Poland; ^3^Department of Epidemiology and Population Studies, Institute of Public Health, Jagiellonian University Medical College, Krakow, Poland; ^4^Department of Social and Preventive Medicine, Medical University of Lodz, Lodz, Poland

## Abstract

The aim of the study was to assess the relationship between the dietary polyphenol intake (DPI) and the dietary total antioxidant capacity (DTAC) and the prevalence of cardiovascular disease (CVD) in postmenopausal women. Participants were 916 postmenopausal women diagnosed with CVD and 1683 postmenopausal women without history of CVD, who took part in the population-based studies carried out in Poland: WOBASZ (2003–2005) and WOBASZ II (2013-2014). Nutritional data were collected using a single 24-hour dietary recall. DPI and DTAC in the CVD women were significantly lower and accounted for 1766.39 mg/d and 10.84 mmol/d, respectively, versus 1920.57 mg/d and 11.85 mmol/d in the women without CVD, but these differences disappeared after the standardization for energy input. Also, in the multiple-adjustment model, higher DPI, but not DTAC, was associated with the reduced odds ratio for the prevalence of CVD. Beverages, mainly coffee and tea, contributed in more than 40% to DPI and in more than a half to DTAC. In this study, higher dietary polyphenol intake, but not the dietary total antioxidant capacity, was inversely associated with CVD in postmenopausal women, which points to the health benefits of increased polyphenol intake from food sources for these women.

## 1. Introduction

Cardiovascular disease (CVD) is the leading cause of mortality in postmenopausal women [1]. Unfavorable behavioral factors during the life as tobacco use, physical inactivity, unhealthy diet, and harmful use of alcohol are fundamental for CVD risk [2]. Deleterious lifestyle patterns translate to the occurrence of intermediate risk factors as raised blood glucose and lipid levels, increased blood pressure, and overweight/obesity [3]. CVD incidence increases with age, and particularly in women, which raises after menopause. Termination of the secretory ovarian function contributes to this morbidity [4]. Because life expectancy has improved and can be up to one-third after menopause and due to increased morbidity and mortality, postmenopausal women should be targeted with all sorts of activities that would reduce the risk of CVD.

At the ground of several chronic diseases that include CVD, diabetes, neurodegenerative diseases (Parkinson's disease and Alzheimer's disease), and cancer and aging is oxidative stress [5]. An excessive generation of extremely reactive oxygen species (ROS) in the body leads to oxidative damage to cellular structures [6]. Some evidence suggests that mechanisms of antioxidant protection can deteriorate with age [7]. Furthermore, heart tissues of elderly people tend to be more vulnerable to oxidative stress, because of the disturbed metabolism in the mitochondria, which leads to oxidative injury [8]. Dietary antioxidants are one of the several lines of defense against cardiovascular disease, diabetes, and cancer. The most important are redox-active dietary constituents as vitamins C and E, carotenoids, and polyphenols [9].

Polyphenols, which are secondary plant metabolites, display potential health effects. The main classes of phenolic compounds include phenolic acids, flavonoids, stilbenes, and lignans [10]. Food products are generally sources of all classes of polyphenols, but they occur in different proportions. The most abundant dietary polyphenols, however, are phenolic acids and flavonoids [11].

The aim of the study was to assess the relationship between the dietary polyphenol intake (DPI) and the dietary total antioxidant capacity (DTAC) and the prevalence of CVD in postmenopausal women and to establish the main dietary sources of polyphenols and antioxidants in postmenopausal women with and without history of CVD.

## 2. Material and Methods

### 2.1. Participants

**Figure 1:**
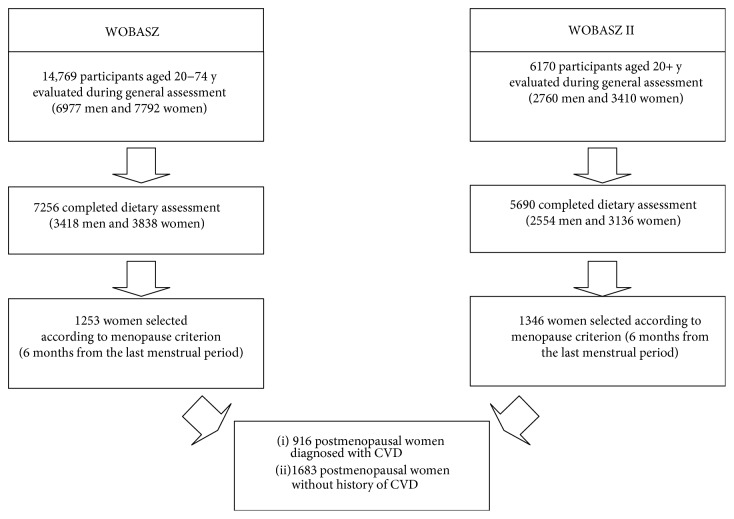
Flow chart of the study participants.

The study examined general, anthropometric, biochemical, and dietary data collected from 2599 postmenopausal women, participants of the two largest population-based cross-sectional studies carried out in Poland: Polish National Multicenter Health Examination Surveys—WOBASZ (2003–2005) and WOBASZ II (2013-2014), which were carried out by the National Institute of Cardiology, Warsaw, Poland, in collaboration with five Polish medical universities. The rationale, design, and methods of the WOBASZ and WOBASZ II studies were described in previous publications [12–14]. Women were selected for the current analysis on the basis of the occurrence of natural menopause and on the basis of completed records. Uniform criteria of six months from the termination of menstruation have been adopted in both WOBASZ studies in accordance with the study manual. The exclusion criteria were pregnancy and surgical menopause. Then, a group of 916 women with medically diagnosed cardiovascular disease (CVD) was extracted from the general study group of postmenopausal women. The remaining women without past history of CVD have served as control. The flow chart of the study participants is provided in Figure 1.

### 2.2. Data Collection

Data on marital status, level of education, menopause status, family history of CVD, history of myocardial infarction or stroke, medications and supplements used, leisure-time physical activity, alcohol intake, and smoking habit were collected from a self-reported standardized questionnaire designed for the WOBASZ study. Cardiovascular disease (CVD) classification was adopted in accordance with the WHO [15]. Hypertension has been recognized if systolic BP ≥ 140 mmHg and/or diastolic BP ≥ 90 mmHg and/or when antihypertensive drugs were used. Criteria for diabetes were glucose level ≥ 7.0 mmol/L and/or the use of glucose-lowering drugs. Two categories of the marital status were included: married and singles, while “singles” were widows, unmarried women, divorced, and separated. Education level was given in three categories: (1) under middle—no education, partial or completed education for primary level, vocational lower secondary education, and partial secondary education; (2) middle—secondary education and partial academic education; and (3) academic education. Smoking status was assessed in three categories: current smokers, past smokers, and never smokers, depending on a habit of smoking at least one cigarette a day. Physical activity at leisure was assessed at a low level—when there was no such physical activity, for example, jogging, cycling, swimming, and gardening for at least 30 minutes a day or only occasional activity (once a week, several times a month, and several times a year); middle level—physical activity, for example, jogging, cycling, swimming, and gardening was for at least 30 minutes a day every second or third day; and high level—physical activity as given above every day or almost every day. Body measurements, such as height, body mass, and waist circumference were taken by the personnel trained in standard procedures. The body mass index (BMI) was calculated as weight in kilograms divided by the square of height in meters. Abdominal obesity has been recognized if waist circumference was >88 cm. Supplementation with at least one of the antioxidant vitamins (A, C, and E) was collected from the self-reported WOBASZ questionnaire. Blood pressure (BP) was measured three times on the right arm after 5 minutes of resting in a sitting position in one-minute intervals, and the final BP was calculated as an average of the second and third measurements. Biochemical analyses, as fasting glucose, total cholesterol, LDL cholesterol, HDL cholesterol, and triglycerides, were carried out at the Central Laboratory of the National Institute of Cardiology, Warsaw. General description of the study group, stratified by CVD status, is given in Table 1.

The WOBASZ study protocol was approved by the Bioethics Committee of the National Institute of Cardiology (WOBASZ, number 708) and (WOBASZ II, number 1344).

### 2.3. Dietary Assessment

Nutritional data were collected by qualified interviewers using a single 24-hour dietary recall. On the basis of the recalls, it was found that 367 dishes, food items, and beverages consumed by the participants were sources of polyphenol intakes. These products were grouped into 5 food categories: beverages (alcoholic and nonalcoholic)—coffee, tea, wine, beer, fruit drinks, and juices; cereals—bread (wheat, rye, and mixed), groats, and macaroni; fruit (fresh, frozen, dried, and jam); vegetables (fresh, frozen, pickled, and legumes); and other food products—cocoa products (chocolates—semisweet chocolate, bitter chocolate, and white chocolate; cocoa), nuts and seeds (almonds, peanut, coconut, hazelnuts, pistachio, walnuts, poppy seeds, sesame seeds, sunflower seeds, and pumpkin seeds), cookies and pastry, and other sweets (candies, etc.). The meal preparation techniques were taken into consideration as factors affecting the polyphenol contents of food items. Individual components of complex dishes have been extracted using dish recipes from the Polish Food Composition Tables [16]. These recipes give the amounts of food items required for 100 g dish portion, with consideration of yield factors. A quantitative composition of plant components of ready-to-eat foods was taken according to information given on food labels. For fruit yoghurt, for example, a typical amount of 5% respective added fruit has been taken for the calculations.

### 2.4. Estimation of Dietary Antioxidant Activity and Polyphenol Intake

Antioxidant activity of food products was mostly taken from our own databases of ferric-reducing antioxidant potential (FRAP) of food [17, 18], which represent mean values of antioxidant activity for 69 food products. Some missing values were complemented with other databases [19]. Dietary antioxidant activity per day as well as daily intakes of polyphenols was determined by multiplying the daily consumption of individual food items by antioxidant activity and polyphenol contents in these food items. The intake of total polyphenols was estimated using our own total polyphenol databases [17, 18] as well as the online Phenol-Explorer database [20]. For the calculation of dietary polyphenol intake (DPI) and the dietary total antioxidant capacity (DTAC) by different databases, the same food items and the same culinary techniques were taken into account.

### 2.5. Statistical Analyses

Descriptive data for the study population characteristics of women diagnosed with CVD and of women without history of CVD were calculated as means and SDs for the continuous variables and as percentages for the categorical variables. For comparison purposes of dietary intakes, Student's *t*-test was used. DTAC and DPI are given unadjusted and adjusted to 1000 kcal to correct for total energy intake. Additionally, all postmenopausal women were grouped into quartiles based on DTAC and DPI total values. Percentages of women diagnosed with CVD were calculated across quartiles of DTAC and DPI. For nonnormally distributed data, such as contributions of food categories to DTAC and DPI, Kruskal-Wallis test was used. Odds ratios (ORs) with the corresponding 95% confidence intervals (CIs) for CVD by DTAC and DPI, unadjusted and adjusted for age, smoking, alcohol intake, education, physical activity, and menopause hormone therapy, were calculated using logistic regression. Due to a wide span between the lowest and the highest value of polyphenol intake, OR for DPI was calculated by 100 units (100 mg/d). All tests of statistical significance were two-sided. The SAS software version 9.2 (SAS Institute Inc., Cary, NC) was used for all the statistical calculations.

## 3. Results

Table 2 displays the mean dietary intakes in the studied postmenopausal women, stratified by the CVD status. It was found that CVD women had statistically lower energy input as well as essential nutrient intakes (protein, carbohydrates, total fat, saturated fat, and cholesterol) compared to the women without CVD. Energy from food was lower in CVD women approximately by 8%, protein by 6%, carbohydrate by 7%, total fat and saturated fat by 11%, and cholesterol by 11.5%. Table 2 also provides the consumption of the most important groups of food products that contain polyphenols, which are vegetables, fruit, tea, and coffee [11]. There were no significant differences in the intake of vegetables, fruits, and tea between the groups of women. However, it was found that women with CVD consumed significantly less coffee. DPI and DTAC, standardized for 1000 kcal of energy, were not significantly different.

Taking into account the raw data, the variables tested in this study, such as DPI and DTAC, were associated with reduced odds of CVD in postmenopausal women (Table 3). Analysis of this data indicates that the women with higher DPI (per 100 units = 100 mg/d) and higher DTAC had 2.3% reduced odds of CVD. Adjustments for multiple variables such as age, smoking, alcohol intake, education, physical activity, and menopause hormone therapy, however, reduced the odds ratio of CVD to 1.1% in the case of DPI, while DTAC was no longer associated with the reduced odds of CVD.

Table 4 shows the percentages of the women with CVD in the individual quartiles of DTAC and DPI, unadjusted as well as adjusted for age, smoking, alcohol intake, education, physical activity, and menopause hormone therapy. The mean values for the lowest versus highest quartiles were as follows: for DPI, it was 948 mg/d versus 2975.8 mg/d and for DTAC, it was 5.05 mmol/d versus 19.87 mmol/l. In the case of both DPI and DTAC, the increasing percent of CVD women within the decreasing DPI and DTAC quartiles was observed. Given the unadjusted data, the least proportion of the women with CVD (29.54%) was observed in the highest quartile of DPI (14.28–191.82 mmol/d) and the largest proportion of the women with CVD (42.06%) was in the lowest quartile of DTAC (range 0.22–7.18 mmol/d). In the case of unadjusted data concerning DPI, the largest percentage of the women with CVD (42.84%) was found in the lowest quartile of DPI (range 1120–1263 mg/d) and the least proportion of the women with CVD (28.15%) was found in the highest quartile of DPI (2303–8793 mg/d). Also in this case, a significant decreasing trend toward the lower percentage of CVD women within the individual increasing DPI quartiles was observed, which was still significant even after the adjustment for age, smoking, alcohol intake, education, physical activity, and menopause hormone therapy. The percentage of CVD women within the individual DTAC quartiles was gradually decreasing. This trend was statistically significant. However, in a multiple-adjustment model, this tendency was no longer significant for DTAC.

Table 5 shows the contributions of individual food categories to the DPI value in the women with and without CVD. The total DPIs in the individual groups were 1766.39 mg/d in the CVD women and 1920.57 mg/d in the women without CVD. These values were statistically significant at *p* < 0.0001. The total DPIs in both study groups were mostly affected by the consumption of beverages, which contributed to the total DPI in 41.81% in the case of the CVD women and in 44.19% in the women without CVD. These differences were statistically significant at *p* < 0.001. In the both groups of women, the coffee and tea consumption accounted for about 98% of the beverages' DPI value. The remaining food categories contributed less to the total DPI value. They were listed according to the decreasing impact: fruits (26.31% in the CVD women and 24.05% in the women without CVD), cereals (14.10% and 13.34%, resp.), vegetables (12.12% and 11.88%, resp.), and other food categories (5.66% and 6.54%, resp.). DPI values from vegetables (*p* = 0.044) and other foods (*p* = 0.0002) were significantly lower in the women diagnosed with CVD. Taking into account the individual food products, given in a descending order, consumption of tea (21.34%), coffee (19.56%), apples (12.79%), potato (5.51%), and mixed bread (4.87%) had the largest impact on DPI in CVD women, as measured by the percentage of the DPI value. Similarly, among women without CVD, the most important foods that influenced DPI were also (in a descending order) coffee (24.34%), tea (18.94%), apples (12.33%), mixed bread (5.59%), and potato (5.56%).

The total and the partial (for each food category) DTAC values for CVD women and the women without CVD are given in Table 6. The total DTAC of 10.84 mmol/d in the women diagnosed with CVD was significantly lower in comparison to that of 11.85 mmol/d in the women without CVD. Beverages were the main food category which contributed in more than a half to the DTAC total value: 53.87% and 57.13% in the women diagnosed with CVD and in the women without CVD, respectively. These differences were statistically significant at *p* < 0.001. Among the beverages, which included alcoholic and nonalcoholic drinks, the consumption of coffee and tea affected the DTAC in the respective groups of women (with CVD and without CVD) in the same extent of 97.8%. The other food categories ranked in terms of impact on the DTAC in the women with CVD versus the women without CVD were fruits, vegetables, other food categories, and cereals. DTAC from the foods grouped in the category of other foods was significantly lower in the CVD women (*p* < 0.0001). It was found that within each of the categories of food, similar foods affected partial DTACs in the both groups of women. For example, apples, strawberries, plums, and grapes delivered most of the DTAC in the fruit food category both in the women with CVD and in the women without CVD. Similar observations were made for cereals, vegetables, and other food categories. With regard to individual food products, a consumption of coffee (26.57%), tea (26.11%), apples (5.81%), potato (5.63%), strawberries (3.69%), and nuts and seeds (3.41%) had the greatest impact on the DTAC in CVD women. Among the women without CVD, the food products, which in the largest percentage influenced on DTAC, were coffee (32.83%), tea (23.04%), potato (5.65%), apples (5.57%), strawberries (3.38%), and nuts and seeds (2.87%).

## 4. Discussion

The results of the present study indicated that polyphenol intake and the antioxidant activity in postmenopausal CVD women were significantly lower and accounted for 1766.39 mg/d and 10.84 mmol/d, respectively, versus 1920.57 mg/d and 11.85 mmol/d in the women without CVD. However, lower energy intake among women with CVD compared to the women without history of CVD led us to make adjustments for energy. After the adjustment to 1000 kcal, both DPI and DTAC did not differ between the groups. Interestingly, in contrast to the current research, in an earlier analysis based on the WOBASZ (2003–2005) study concerning CVD women of different ages, an increase in dietary antioxidant activity and a greater consumption of polyphenols than those in the healthy women were observed, which was explained by healthier dietary choices by CVD women [21]. In contrast, the present study shows dietary modifications made by postmenopausal CVD women in terms of reduced calorie and coffee intakes, but the consumption of vegetables and fruits was at a similar level. These modifications were reflected in the DPI and DTAC values. At the same time, the study indicates (after the adjustment of model for multiple variables as age, smoking, alcohol intake, education, physical activity, and menopause hormone therapy) that there was a small reduction observed with regard to DPI, but no decline in the odds of CVD in the women according to DTAC. In addition, it was found that coffee and tea were the main sources of DPI and DTAC in the studied postmenopausal women, which is consistent with other observations in Polish urban population [22].

Senescence is characterized by increased rates of hypertension, diabetes, CVD, and cancer [23]. Several studies found an inverse relationship between dietary total antioxidant capacity (DTAC) and the risk of chronic diseases in aging people. A meta-analysis of those data concluded that DTAC has a great potential for clinical applications and public health [24]. Previously, we found a diminished DTAC value in an elderly population, and particularly in the elderly women, compared to that in the young and middle-aged subjects [25]. DTAC, among other things, corresponds with polyphenol content in the diet. It has not been established, however, whether the effect of polyphenols on cardiovascular health is directly related to their antioxidant activity [26]. Several other mechanisms of polyphenol action in humans, which are not related to direct antioxidant activity, have been suggested, including microbiota transformation or the enhancement of internal mechanisms of antioxidant protection [27, 28]. Regardless of the mechanisms of action, beneficial polyphenol effects on the heart health were observed. In the PREDIMED study, a 46% reduction in the risk of CVD during 4.3 years of a follow-up period was observed, comparing Q5 with Q1 of total polyphenol intake [29]. Recently, we found that the existing databases of dietary polyphenols are partially incomplete and should be further expanded to better reflect the dietary polyphenol contents in foods [11]. Therefore, on the basis of our own dietary database [17, 18] as well as of other available published databases [20] and taking into account the amounts of individual food consumed, we calculated dietary polyphenol intakes (DPI) for postmenopausal women with CVD and the non-CVD women. Likewise, dietary total antioxidant capacity (DTAC) was calculated using our own [17, 18] and other commonly accessible databases [19], which are based on the FRAP (ferric-reducing antioxidant potential) assay. At this point, it should be mentioned that the research methodology of dietary TAC is varied. The FRAP assay is the most widely used experimental method to assess TAC in foods, although several methods have been developed for measuring total antioxidant capacity [27]. Several studies demonstrate that higher TAC from diet and supplements beneficially alters lipid and glucose profiles [30, 31], improves endothelial function [32], and reduces systemic inflammation [33]. Recent clinical studies have shown that higher TAC from diet and supplements can be associated with a decrease in inflammatory markers in overweight/obese postmenopausal women [34]. Supplements in this study largely contributed to the TAC value. Conversely, reduced rates of CVD were not observed for a single antioxidant vitamin supplement (beta-carotene, vitamin C, and vitamin E) users in randomized controlled trials [35, 36]. In contrast to this research, supplementation in our study was not calculated, with reference to the purpose of the study, which was to demonstrate a dietary impact on TAC. In addition to this, in various studies, DTAC has been calculated with different methods. DTAC in our own study was determined by using the experimental FRAP method that measures combined TACs of food samples [27]. It is common to use the FRAP assay in order to assess the relationship between the dietary TAC and the occurrence of diseases. In several studies, a negative association between the dietary TAC and the occurrence of cancers has been found [37, 38], although not all studies support the existence of such dependency [39]. Another approach is to determine DTAC on the basis of theoretical calculations of individual antioxidants (polyphenols, carotenoids, and vitamins C and E) in food products. In our study, however, TAC value of individual food products results from a complex interaction of many of the components included in one food sample. We found, however, that diets with higher TAC, after adjustment for potential confounders for CVD in postmenopausal women such as age, smoking, alcohol intake, education, physical activity, and menopause hormone therapy, in contrast to DPI, were not associated with the reduced odds of CVD. Possibly, the content of antioxidants in the diet is insufficient to significantly affect the level of antioxidants in the human body. Generally, despite polyphenol dietary abundance, their bioavailability is rather low [26]. Therefore, other mechanisms for their actions are suggested, as it was mentioned before. In addition, bioavailability of some other food ingredients, such as carotenoids, for example, can be negatively affected by the digestion and absorption process [40].

Quartile analysis provides more information on the relationship between DTAC and DPI and the prevalence of CVD. Despite that DTAC in the upper quartile is almost four times higher than that in the bottom quartile, the number of women with CVD in the upper quartile is only about 17% lower in the model of multiple adjustments than that in the bottom quartile, and this trend is not significant. A similar situation applies to DPI, which in the upper quartile is three times higher than that in the lower quartile, while the number of women with CVD in the upper quartile is only ~22% lower than that in the bottom quartile. However, in the case of DPI, this trend is statistically significant, showing that the total polyphenol intake may affect the incidence of CVD only to a small extent, while other factors such as lifestyle-related factors, may be relevant to CVD risk in postmenopausal women. It is also possible that only an in-depth analysis of individual polyphenols could give the answer to the argument raised in this paper, but this requires further analysis.

By comparing the composition of the diets in the both groups of women, we found that the supply of energy, protein, carbohydrates, and fats was significantly lower in the women with CVD. Lower energy and nutrient intakes might have resulted somewhat from both an older age of the CVD women and possibly due to the nutritional restrictions because of the increased body mass as well as larger prevalence of diabetes in the group of CVD women. CVD women in this study were characterized by a higher proportion of obesity cases [44.3%) and diabetes (19.8%) compared to non-CVD women (32.2% and 13.4%, resp.). The prevalence of overweight and obesity is common in postmenopausal women in the Polish population [41], and an excessive body weight is associated with oxidative stress [42]. We also found that despite the lower energy and nutrient intakes from the diet, the supply of vegetables and fruits in the postmenopausal CVD women remained similar to that in the women without CVD. Vegetables and fruit are together a group of food that was consumed in the highest amounts (together, they represent the amount of 430 g/d). They were one of the main sources of antioxidants and polyphenols in the both groups of women. Also, the structure of food consumed by the participants was similar in the respective groups of women. The main beverages in either case were coffee and tea. The tea intake did not differ significantly between the two groups of women, while the consumption of coffee was significantly lower among CVD women. Some alcoholic beverages, such as wine and beer, which normally are polyphenolic supplies for the general population, had no impact on the DPI and DTAC values in the studied group of postmenopausal women.

The results concerning the intake of coffee and the risk of CVD are conflicting. One of the most important risk factors of CVD is high blood pressure. In hypertensive elderly populations, a habitual coffee drinking may lead to uncontrolled blood pressure (BP) [43]. However, several cohort studies and a meta-analysis do not confirm an association between longer-term coffee consumption and increased BP and also between habitual coffee consumption and the increased risk of CVD in hypertensive subjects [44]. In other study in Poland, it was indicated that the risk of hypertension was lower in persons consuming moderate amount of coffee (3-4 cups/day) and higher intake of coffee was not protective [45]. A literature search shows that moderate intake of coffee appears to be protective against CVD [46–49] and coffee consumption can confer benefits, particularly for postmenopausal women, by reducing inflammation and the risk of CVD [50]. Recent studies demonstrate that drinking 1-2 cups of coffee per day in women reduces the risk of mortality due to CVD, and this effect was observed in nonsmokers only [46]. Interestingly, DPI and DTAC from the remaining food categories did not differ in principle, with the exception of the category of other foods (for DPI and DTAC) and vegetables (for DPI). But overall, the beverages had a determining influence on the total values of the DPI and DTAC. In addition, the analysis of the structure of the DTAC and DPI in various food categories indicates similar selection of food products in the both groups of women (with CVD and without CVD).

This study has its strengths and limitations. The advantage of this population-based study is a large group of postmenopausal women; however, the participation rate in the WOBASZ II study was rather low. Also, this study provides data regarding the relationship between DTAC and the prevalence of CVD in postmenopausal women, which was not so far studied. Among the limitations, first is the cross-sectional nature of the study which does not allow to present time sequence between the disease and exposure. The results may be biased by the reverse causality; persons after the diagnosis of CVD could have been advised to change the diet and to decrease the consumption of coffee. Next, a single 24-hour recall does not reflect the long-term consumption. Therefore, small dietary variations of the examined women might have affected the results obtained. However, there is no satisfactory tool to evaluate food consumption, and each of these methods has its strengths and weaknesses [51]. The single 24-hour recall method was used in WOBASZ and WOBASZ II studies, because in contrast to other methods, it is simple, inexpensive, and relatively brief, with less potential to interfere with respondent's dietary behavior and usually with a better response rate which translates into increased representativeness of the population. For these reasons, it is often used in epidemiological studies. Possibly, the use of multiday recalls would be more beneficial, because they give better estimates of the population's dietary intake [51], but there is more burden on the respondents. Another alternative for the 24-hour recall is a food frequency questionnaire. This latter, however, does not measure many details of dietary intake, which is essential for the good interpretation of dietary intake of polyphenols, which are present in a variety of foods. For the above reasons, a single 24-hour recall method was selected.

## 5. Conclusion

In this study, higher dietary polyphenol intake, but not the dietary total antioxidant capacity, was found to be inversely associated with CVD in postmenopausal women, which points to the health benefits of increased polyphenol intake from food sources for these women.

## Figures and Tables

**Table 1:** General description of the studied postmenopausal women with and without CVD.

Trait	Women diagnosed with CVD *N* = 916	Women without CVD *N* = 1683	Significance level
	Mean ± SD or %	Mean ± SD or %	*p*
Age (years)	65.5 ± 9.2	60.98 ± 8.4	<0.0001
Marital status (%)			
Married	57.2	63.9	0.001
Single	42.8	36.1	
Level of education (%)			
Under middle	61.0	55.2	0.009
Middle	31.9	35.4	
Academic	7.1	9.5	
Age of natural menopause (years), mean, SD, range	49.9 ± 4.4	50.0 ± 4.1	0.875
Duration of menopause (years), mean, SD, range	15.6 ± 9.6	11.0 ± 8.6	<0.0001
Family history of CVD (%)	39.4	36.1	0.098
Diseases (%)			
Hypertension	73.1	57.3	<0.0001
Myocardial infarction	12.6	0	<0.0001
Stroke	7.9	0	<0.0001
Diabetes	19.8	13.4	<0.0001
Medication			
Menopause hormone therapy (%)	3.8	4.9	0.178
Hypotensive drugs (%)	61.0	34.9	<0.0001
Cholesterol-lowering therapy (%)	29.3	13.5	<0.0001
Antidiabetic medication or insulin (%)	15.6	8.2	<0.0001
Smoking status (%)			
Current smokers	12.5	20.9	<0.0001
Past smokers	18.6	17.7	
Never smokers	68.9	61.4	
Leisure-time physical activity (%)			
Low level	52.1	47.8	0.107
Middle level	16.5	18.4	
High level	31.3	33.8	
BMI (kg/m^2^) (%)			
Underweight (BMI < 18.5)	0.3	0.7	<0.0001
Normal (BMI 18.5–24.99)	28.2	27.8	
Overweight (BMI 25–29.99)	35.2	39.2	
Obesity (BMI > 30)	44.3	32.2	
Abdominal obesity (%)	71.1	62.5	<0.0001
Supplementation with antioxidant vitamins (A, C, and E) (%)	9.9	8.9	0.391
Alcohol intake (g pure ethanol/day), mean, SD	0.7 ± 2.7	1.0 ± 2.9	<0.0001
Fasting glucose (mmol/l), mean, SD	5.7 ± 1.9	5.5 ± 1.6	0.531
Total cholesterol (mmol/l), mean, SD	5.4 ± 1.2	5.8 ± 1.3	<0.0001
LDL cholesterol (mmol/l), mean, SD	3.2 ± 1.1	3.6 ± 1.1	<0.0001
HDL cholesterol (mmol/l), mean, SD	1.4 ± 0.4	1.5 ± 0.4	<0.0001
Triglyceride (mmol/l), mean, SD	1.6 ± 1.0	1.5 ± 0.8	0.1006

**Table 2:** Mean dietary intakes in the postmenopausal women by prevalence of CVD.

Consumption	Women diagnosed with CVD *N* = 916	Women without CVD *N* = 1683	
	Mean ± SD	Mean ± SD	*p*
Energy from food (kcal/d), mean, SD	1516.9 ± 579.5	1652.9 ± 628.1	<0.0001
Protein (g/d), mean, SD	55.6 ± 22.7	58.9 ± 23.6	<0.0001
Carbohydrate (g/d), mean, SD	209.1 ± 86.2	224.3 ± 90.1	<0.0001
Total fat (g/d), mean, SD	57.7 ± 28.7	64.9 ± 31.9	<0.0001
Saturated fat (g/d), mean, SD	21.7 ± 11.9	24.4 ± 13.0	<0.0001
Cholesterol (mg/d), mean, SD	207.1 ± 140.0	234.2 ± 153.7	<0.0001
Vegetables (g/d)	215.0 ± 156.0	214.0 ± 155.0	0.850
Fruits (g/d)	214.0 ± 226.0	216.0 ± 227.0	0.989
Tea (g/d)	360.9 ± 251.7	348.1 ± 250.6	0.180
Coffee (g/d)	129.6 ± 165.5	175.3 ± 171.9	<0.0001
DPI (mg/d/1000 kcal)	1243.0 ± 654.0	1236.0 ± 528.0	0.493
DTAC (mmol/d/1000 kcal)	7.7 ± 5.6	7.7 ± 4.6	0.099

**Table 3:** Odds ratios (ORs) and 95% confidence intervals (CIs) for prevalence of CVD by dietary total antioxidant capacity (continuous) and dietary polyphenol intake (per 100 mg/d).

Variables	Model 1^a^ OR (95% CI)	Model 2^b^ OR (95% CI)
DPI (mg/d)	0.977 (0.968; 0.987)	0.989 (0.978; 0.999)
DTAC (mmol/d)	0.977 (0.964; 0.990)	0.992 (0.979; 1.005)

^a^Unadjusted. ^b^Adjusted for age, smoking, alcohol intake, education, physical activity, and menopause hormone therapy.

**Table 4:** Percentages of CVD women, odds ratios (ORs), and 95% confidence intervals (CIs) for prevalence of CVD by quartiles of DTAC and DPI.

Parameters		Quartile 1	Quartile 2	Quartile 3	Quartile 4	*p*
DPI (mg/d)	Mean ± SD	948.2 ± 236	1523.2 ± 142	2016.3 ± 154	2975.8 ± 724	
	Range	(1120–1263)	(1264–1759)	(1760–2302)	(2303–8793)	
	% CVD^a^	42.84	36.00	34.00	28.15	<0.0001
	% CVD^b^	40.60	35.04	34.50	31.80	0.011
DTAC (mmol/d)	Mean ± SD	5.05 ± 1.50	8.84 ± 0.96	12.20 ± 1.05	19.87 ± 9.74	
	Range	(0.22–7.18)	(7.19–10.48)	(10.49–14.27)	(14.28–191.82)	
	% CVD^a^	42.06	36.31	33.08	29.54	<0.0001
	% CVD^b^	39.72	34.87	34.12	33.17	0.073

^a^Unadjusted. ^b^Adjusted for age, smoking, alcohol intake, education, physical activity, and menopause hormone therapy.

**Table 5:** Contributions of food categories to DPI in the women with and without CVD.

Food categories		Women with CVD *N* = 916	Women without CVD *N* = 1683	*p* value
Beverages	mg/d (mean ± SD)	738.47 ± 471	848.61 ± 492	<0.001
	Contribution to DTAC (%)	41.81	44.19	
	Major sources (% contribution)^∗^	Tea (21.34), coffee (19.56)	Coffee (24.34), tea (18.94)	
Cereals	mg/d (mean ± SD)	249.01 ± 150	256.17 ± 157	0.188
	Contribution to DTAC (%)	14.10	13.34	
	Major sources (% contribution)^∗^	Mixed bread (4.87), rye bread (3.36), wheat bread (3.20)	Mixed bread (5.59), pastry (2.83), rye bread (2.91)	
Fruit	mg/d (mean ± SD)	464.72 ± 519	461.86 ± 499	0.735
	Contribution to DTAC (%)	26.31	24.05	
	Major sources (% contribution)^∗^	Apples (12.79), plums (3.61), strawberries (2.25)	Apples (12.33), plums (2.75), strawberries (2.12)	
Vegetables	mg/d (mean ± SD)	214.09 ± 147	228.23 ± 156	0.044
	Contribution to DTAC (%)	12.12	11.88	
	Major sources (% contribution)^∗^	Potato (5.51), tomato (1.49), cabbage (1.39)	Potato (5.56), cabbage (1.33), tomato (1.30)	
Other food categories	mg/d (mean ± SD)	100.10 ± 199	125.70 ± 208	0.0002
	Contribution to DTAC (%)	5.66	6.54	
	Major sources (% contribution)^∗^	Cookies and pastry (2.50), cocoa products (2.08), nuts and seeds (0.81)	Cookies and pastry (2.83), cocoa products (2.53), nuts and seeds (0.86)	
Total	mg/d (mean ± SD)	1766.39 ± 865	1920.57 ± 825	<0.0001
	Contribution to DTAC (%)	100	100	

^∗^In each food category, individual food products with the strongest impact on the DPI were only listed.

**Table 6:** Contributions of food categories to DTAC in the women with and without CVD and major dietary sources that impact DTAC within each food category.

Food categories		Women with CVD *N* = 916	Women without CVD *N* = 1683	*p* value
Beverages	mmol/d (mean ± SD)	5.84 ± 3.84	6.77 ± 4.03	<0.001
	Contribution to DTAC (%)	53.87	57.13	
	Major sources (% contribution)^∗^	Coffee (26.57), tea (26.11)	Coffee (32.83), tea (23.04)	
Cereals	mmol/d (mean ± SD)	0.38 ± 0.29	0.39 ± 0.27	0.206
	Contribution to DTAC (%)	3.51	3.29	
	Major sources (% contribution)^∗^	Mixed bread (1.11), rye bread (0.83), wheat bread (0.74)	Mixed bread (1.27), rye bread (0.68), wheat bread (0.59)	
Fruit	mmol/d (mean ± SD)	2.19 ± 3.48	2.11 ± 3.05	0.427
	Contribution to DTAC (%)	20.20	17.81	
	Major sources (% contribution)^∗^	Apples (5.81), strawberries (3.69), plums (2.03), grapes (1.94)	Apples (5.57), strawberries (3.38), plums (1.6), grapes (1.43)	
Vegetables	mmol/d (mean ± SD)	1.67 ± 1.58	1.77 ± 1.62	0.124
	Contribution to DTAC (%)	15.41	14.94	
	Major sources (% contribution)^∗^	Potato (5.63), beetroot (2.77), cabbage (1.85), tomato (1.11), broccoli and cauliflower (0.92)	Potato (5.65), beetroot (2.70), cabbage (1.94), broccoli and cauliflower (1.01), tomato (0.93)	
Other foods	mmol/d (mean ± SD)	0.76 ± 5.82	0.81 ± 3.46	<0.0001
	Contribution to DTAC (%)	7.01	6.83	
	Major sources (% contribution)^∗^	Nuts and seeds (3.41), cocoa products (1.85), cookies and pastry (0.73)	Nuts and seeds (2.87), cookies and pastry (2.87), cocoa products (1.94)	
Total	mmol/d (mean ± SD)	10.84 ± 8.53	11.85 ± 6.66	<0.0001
	Contribution to DTAC (%)	100	100	

^∗^In each category of food products, only those that had the greatest impact on the DTAC were listed.
